# Predictors of depressive symptoms and depression in women with previous pregnancy loss

**DOI:** 10.1111/bjc.70032

**Published:** 2025-12-28

**Authors:** Sarah M. Quaatz, Roland Mergl, Franziska Bichlmayer, Helena Hoffmann, Kathryn Eichhorn, Antje‐Kathrin Allgaier, Svenja Hoffmann

**Affiliations:** ^1^ Institute of Psychology University of the Bundeswehr Munich Neubiberg Bavaria Germany

**Keywords:** depression, depressive symptoms, miscarriage, predictors, pregnancy loss, stillbirth

## Abstract

**Objectives:**

Depressive symptoms are common in females after pregnancy loss. However, research on risk factors for developing a clinical depressive episode remains limited and with inconsistent findings. This study examined the association of demographic variables (like age), pregnancy‐related factors (like number of miscarriages or stillbirths), clinical factors (like medical explanation for the pregnancy loss) and neuroticism (as a well‐known risk factor for internalizing disorders) with a current major depressive episode and depression severity in 172 women with miscarriage or stillbirth within the previous year.

**Design:**

Case–control study.

**Methods:**

Measures included the semi‐structured diagnostic interview DIPS, the State–Trait Anxiety‐Depression Inventory (STADI), the Short Version of the Big‐Five Inventory (BFI‐K). To identify predictors of depression, linear and logistic regression analyses were conducted.

**Results:**

Lower medical support (*p* = .041), higher stress immediately after the pregnancy loss (*p* = .027) and higher neuroticism scores (*p* = .008) were significant predictors of a current major depressive episode in woman with pregnancy loss. Incoherent farewell (*p* = .043), a history of psychotherapy or psychiatric treatment in the last year (*p* = .016) and higher neuroticism scores (*p* < .001) were significantly associated with higher severity of depressive symptoms.

**Conclusions:**

Methodological strengths of our study include a well‐validated clinical interview and the investigation of the whole spectrum of gestation age at the time of pregnancy loss. Longitudinal studies should be used to replicate our findings. The knowledge of significant predictors is relevant for the identification of high‐risk groups for depression among women with pregnancy loss.

**Practitioner points**

- Knowledge of low medical support, high stress immediately after pregnancy loss and high neuroticism is relevant for identifying high‐risk groups for the development of a depressive episode among women after pregnancy loss.
- An incoherent farewell, a history of psychotherapy/psychiatric treatment in the last year and higher neuroticism go along with more pronounced depressive symptoms in women with pregnancy loss.

## INTRODUCTION

Pregnancy loss is a common phenomenon with approximately 23 million miscarriages occurring each year (Quenby et al., 2021) and about 2 million stillbirths (UNICEF, WHO, WBG, UN, 2020). For both kinds of pregnancy loss, the detrimental effects on mental health can be pronounced (Burden et al., 2016; Farren et al., 2018; Quenby et al., 2021). Miscarriages and stillbirths represent severe loss events and may be linked to depression (Mergl et al., 2024). Therefore, assessing the risk of developing depression after a miscarriage or stillbirth is of particular interest.

Studies on depressive symptoms and depressive disorders in women after miscarriage and stillbirth have so far allowed few conclusions, often due to different inclusion and exclusion criteria. However, a very recent review by Mergl et al. (2024) showed that women with previous pregnancy loss have an increased risk of developing depressive symptoms and disorders. The range regarding prevalence of depressive symptoms was very wide (5–91.2%) and the prevalence of depressive disorders had a range of 5.4–18.6%. Longitudinal studies show that most of the women affected have elevated depression scores in the first few months after the loss and that the symptoms subside in some women after some time (Farren et al., 2018; Mergl et al., 2024).

On the basis of these results, several risk factors have been discussed as predictors of depressive symptoms and depressive disorders in women after pregnancy loss (e.g., deMontigny et al., 2020; Farren et al., 2018; Farren et al., 2022; He et al., 2019; Kukulskienė & Žemaitienė, 2022; Mutiso et al., 2018; Westby et al., 2021). Table S1 provides an initial detailed overview of more recent studies in which predictors of depressive symptoms and/or depressive disorders in women after pregnancy loss were examined. Without making any claim to completeness, the previous literature on the most promising predictors can be summarized as follows (for information on other predictors, see Table S1):

Regarding the *number of miscarriages or stillbirths*, a recent meta‐analysis concerning depression outcomes in women with recurrent spontaneous abortion (≥3) suggests a higher number of spontaneous abortions to be significantly associated with the occurrence of depressive symptoms in this group (Zhang et al., 2024). According to another recent systematic review and meta‐analysis, recurrent perinatal loss (≥2) was found to have the largest effect on depressive symptoms, followed by neonatal death, stillbirth, miscarriage, termination of pregnancy due to foetal anomaly (TOPFA) and grouped pregnancy loss (Herbert et al., 2022). A narrative review by Farren et al. (2018) identified miscarriage as a risk factor for depressive symptoms in females with early pregnancy loss. The same was true for perinatal loss in women with stillbirths according to the systematic review and meta‐analysis by Burden et al. (2016). Furthermore, a very large cohort study in the UK Biobank with nearly 180,000 female participants indicated an increasing risk for the occurrence of depressive disorders according to ICD‐10 (WHO, 1992) diagnostic criteria with the number of spontaneous miscarriages (Hu et al., 2024), compared to women without a history of spontaneous miscarriages.

For the *kind of pregnancy loss*, stillbirths were associated with a larger risk for depressive symptoms than miscarriages (Herbert et al., 2022). However, the corresponding relative risk differences failed to be statistically significant. In contrast, a recent study by Balle et al. (2024) found that the risk for depressive symptoms was significantly higher in women with stillbirths compared to women having had miscarriages within the last 12 months. According to the narrative review by Farren et al. (2018), longer gestation in women with miscarriages was a significant predictor of later developing depressive symptoms, suggesting differences between women with early versus late miscarriages. Overall, findings on the gestation duration as a potential risk factor for depression in women with pregnancy loss remain inconsistent. Some studies reported positive findings (e.g., Mutiso et al., 2019; Obi et al., 2009), whereas others found no significant effects, (e.g., Broen et al., 2006; He et al., 2019; Kukulskienė & Žemaitienė, 2022; Sham et al., 2010). So far, only one study (Farren et al., 2022) has compared the risk of developing depressive symptoms one month after an early pregnancy loss among women with miscarriages versus ectopical pregnancy versus other forms of an early pregnancy loss. The corresponding group differences were not significant.

Considering a *coherent farewell from the deceased embryo or foetus* (i.e., whether the woman was able to say goodbye in a manner that felt appropriate to her), only a few studies have so far investigated this potential risk factor for depression after pregnancy loss: Research on stillbirths focused on contact with the baby after the pregnancy loss shows that higher depression scores are found in women who had less contact than desired (Campbell‐Jackson & Horsch, 2014). Similarly, another review (Westby et al., 2021) confirmed this by showing that spending less time with the baby than desired was a risk factor for depressive symptoms in women with stillbirths. In line with these findings, Burden et al. (2016) identified ambivalence regarding burial arrangements, as well as not seeing, holding or saying goodbye to the baby, as risk factors for the development of depressive and other negative psychological symptoms in women with stillbirths. However, corresponding findings for miscarriages are still lacking.

*Medical support* has been identified as a protective factor for depressive symptoms in women with stillbirths (Burden et al., 2016; Westby et al., 2021). In contrast, insensitive treatment by healthcare professionals has been shown to be a risk factor for the development of depressive and other negative psychological symptoms in women with stillbirths (Burden et al., 2016). For women with miscarriages, the procedure following abortion (categorized as: no intervention; medical treatment (evacuation with misoprostol); or surgical evacuation by uterine curettage) was not a risk factor for depressive disorders (Sham et al., 2010). Similarly, the mode of treatment of the miscarriage (expectant (missed abortion), medical, or surgical) was neither a risk nor a protective factor for depressive symptoms in this group (Mutiso et al., 2019). There is contradictory evidence regarding low satisfaction with health care in women with miscarriages: Whereas deMontigny et al. (2020) identified low satisfaction as a risk factor for depressive symptoms in women with spontaneous abortions within the past 4 years (even after adjustment for covariables), they found no significant association between dissatisfaction with healthcare services and depressive symptoms in women with at least one miscarriage in the past 6 years in an earlier study (deMontigny et al., 2017).

Beyond these factors, the *occurrence of complications in the index pregnancy* may mark a subgroup at elevated risk for depressive symptoms, at least among women with stillbirths. In the stillbirth literature, obstetric complications around the loss have been linked to higher levels of depressive and other adverse psychological outcomes (Burden et al., 2016; Westby et al., 2021). Whether a comparable association exists for miscarriage remains uncertain, as evidence is sparse and heterogeneous (see Table S1).

The role of *stress immediately after the pregnancy loss* as a risk factor for depression in women after miscarriage or stillbirth has rarely been investigated: A study by Kukulskiene and Zemaitiene (2022) found that poorer physical and emotional well‐being immediately after a miscarriage were associated with an increased risk for depressive symptoms in females with at least one prior miscarriage. However, whether this is also true for women with stillbirths has not yet been examined.

Concerning *maternal age*, younger women seem to be at higher risk of depression after miscarriages (for review, see Farren et al., 2018). However, evidence for this is conflicting. While Sham et al. (2010) and Hu et al. (2024) identified younger age as a risk factor for depressive disorders after miscarriages, and Kukulskiene and Zemaitiene (2022) found younger age to be associated with depressive symptoms after miscarriages, other studies report no significant association between maternal age and subsequent depressive symptoms after miscarriages (Broen et al., 2006; Farren et al., 2022). A recent meta‐analysis (Zhang et al., 2024) concluded that maternal age was not associated with an increased risk for depressive symptoms in women with recurrent spontaneous abortion. Similarly, maternal age did not appear to influence depressive risk in women with perinatal loss, including miscarriage, stillbirth, TOPFA or neonatal death (for review, see Herbert et al., 2022). However, women with stillbirths present a different picture: In this group, higher maternal age was found to be significantly associated with an increased risk for subsequent depressive and other negative psychological symptoms (for review, see Burden et al., 2016).

With respect to *educational level*, Westby et al. (2021) mentioned in their systematic review that educational status was only a weak to moderate predictor of depressive disorders in women with pregnancy losses. In this context, higher education seems to be a protective factor associated with lower depression scores (for review, see Campbell‐Jackson & Horsch, 2014). However, it should be noted that both (Campbell‐Jackson & Horsch, 2014; Westby et al., 2021) investigated only women with stillbirths, making it difficult to generalize these findings to other kinds of pregnancy loss. For other kinds of pregnancy loss, the evidence differs: A recent meta‐analysis (Zhang et al., 2024) showed that educational level was not significantly associated with the risk for depressive symptoms in women with recurrent spontaneous abortion. In women with miscarriages, the corresponding evidence remains inconsistent: Whereas two recent studies reported that lower educational levels were significantly associated with an increased risk for depression (Kukulskienė & Žemaitienė, 2022; Mutiso et al., 2019), other studies found no significant association (Broen et al., 2006; Farren et al., 2022; Sham et al., 2010).

As for the *presence of living children*, childlessness has been identified as a risk factor for depressive symptoms in women with early pregnancy loss (for review, see Farren et al., 2018) although there is also negative evidence (e.g., Farren et al., 2022; Wang et al., 2023). Similarly, the absence of living children was associated with an elevated risk for depressive symptoms in women with stillbirths (Burden et al., 2016). Consistent with this finding, previous and subsequent live births were negatively correlated with depression scores in women with stillbirths (Campbell‐Jackson & Horsch, 2014). Moreover, live birth was also a protective factor regarding depressive symptoms in women with recurrent spontaneous abortion (for review, see Zhang et al., 2024).

With reference to *partnership*, dissatisfaction with emotional partner support after stillbirth was associated with higher depression scores (Campbell‐Jackson & Horsch, 2014). The same was true for poor support from the partner after a stillbirth (Burden et al., 2016). Among women with early pregnancy loss, low marital satisfaction or support were associated with an elevated risk for depressive symptoms (Farren et al., 2018). However, there were negative corresponding findings, too (e.g., Sham et al., 2010).

*Neuroticism* is a robust transdiagnostic risk factor for psychopathology, including depression. An individual‐participant meta‐analysis across 10 cohorts shows higher neuroticism to be associated with higher depressive symptom levels (Hakulinen et al., 2015). Evidence specific to pregnancy loss remains limited: A narrative review with women after early pregnancy loss emphasize background risk factors such as prior psychiatric history and low social support (Farren et al., 2018). Furthermore, a significant positive association between neuroticism and concurrent depression in women following miscarriage was found (Farren et al., 2018). Neuroticism could be linked to heightened stress reactivity, negative affectivity and rumination, which may amplify the psychological impact of loss and hinder recovery. Moreover, neuroticism was found to be significantly associated with a higher genetic risk for depression (Gupta et al., 2024; Kendler & Myers, 2010).

What women are told about the cause of the pregnancy loss may shape cognitive appraisals, self‐blame and uncertainty. A clear *medical explanation for the pregnancy loss* that locates the cause in largely uncontrollable factors (e.g., foetal genetic anomalies) could buffer guilt, whereas absent or ambiguous explanations may maintain distress. Both, feelings of personal guilt and elevated distress can facilitate the development of depression in predisposed women after pregnancy loss; thus, it seems to be relevant to consider the medical explanations for the pregnancy loss as possible risk factor for depression in women with pregnancy losses. To our knowledge, no study has directly tested ‘medical explanations’ as a predictor of depression after miscarriage or stillbirth so far.

The same is true for the variables ‘stress during pregnancy’ and ‘history of psychotherapy or psychiatric treatment in the last 12 months’ although it can be hypothesized that stress during pregnancy (in particular, chronic stress) triggers depression and that a history of psychotherapy or psychiatric treatment within the previous year signifies a subgroup of women being prone to the development of depressive symptoms or disorders after a major critical life event such as pregnancy loss.

In summary, the marked differences between study findings regarding predictors of depression in women with pregnancy loss require further investigation. The existing literature reveals that more research on corresponding risk and protective factors is urgently needed (e.g., Campbell‐Jackson & Horsch, 2014). In addition, methodological differences and limitations across studies continue to make it difficult to derive clear findings on the predictors of depressive symptoms and disorders in women after miscarriages and stillbirths. For instance, findings on prevalence rates, e.g., as collected through standardized diagnostic interviews, are rare. As a result, it remains unclear which predictors could effectively identify high risk populations to offer specialized care interventions and mental health services immediately or to prevent the symptoms from becoming chronic. Thus, the present study aims to identify possible risk and protective factors for both current depressive disorders and symptom severity in women who have experienced miscarriage and stillbirths within the last 12 months. To achieve this, the study utilizes both a questionnaire and a standardized diagnostic interview.

## MATERIALS AND METHODS

### Study design and participants

**FIGURE 1:**
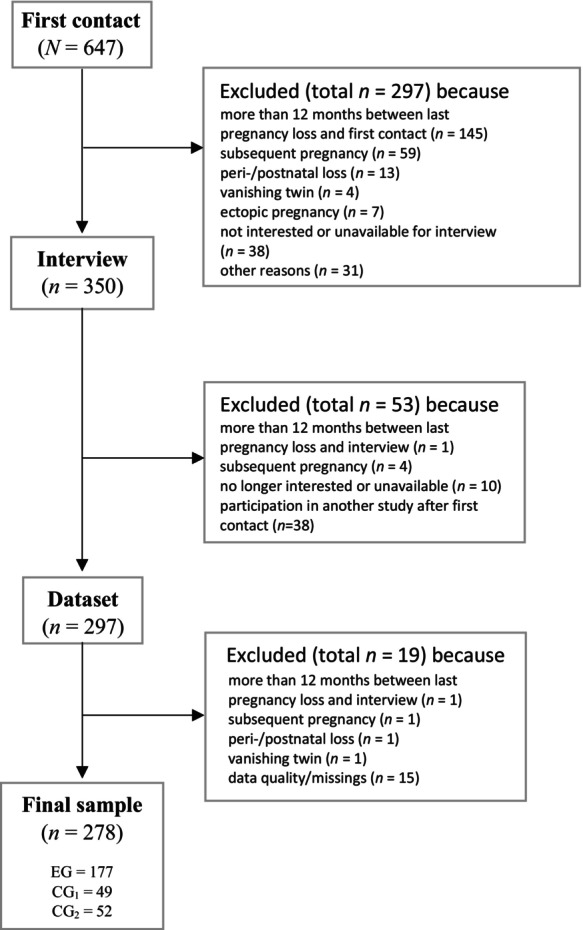
Flow‐chart of participants. EG, examination group consisting of 177 women who had suffered a pregnancy loss within the last 12 months (women with miscarriage (<24th week of pregnancy and <500 g weight of the baby lost), women with stillbirth (≥24th week of pregnancy or ≥500 g weight of the baby lost) and women after medically indicated abortion) but no subsequent pregnancy since the pregnancy loss had occurred. CG = control group: Women with a childbirth within the last 12 months and no history of pregnancy loss were assigned to CG_1_. CG_2_ comprised women with at least one living child aged 1–17 years living in their household but without a pregnancy loss in their history and without a childbirth within the last year.

In our cross‐sectional study with case control group design, we recruited *N* = 647 women for the screening. General inclusion criteria for all groups were female gender, sufficient German language knowledge and age between 18 and 50 years. The examination group consisted of women who had suffered a pregnancy loss within the last 12 months (women with miscarriage (<24th week of pregnancy and <500 g weight of the baby lost), women with stillbirth (≥24th week of pregnancy or ≥500 g weight of the baby lost) and women after medically indicated abortion) but no subsequent pregnancy since the loss had occurred. Women with a childbirth within the last 12 months and no history of pregnancy loss were assigned to control group 1. Control group 2 comprised women with at least one living child aged 1–17 years living in their household but without a pregnancy loss in their history and without a childbirth within the last year. This manuscript reports a secondary analysis of the case–control data set, in which only the examination group (*n*_EG_ = 177) is considered. Figure 1 demonstrates the reasons for exclusion from the sample after screening, ultimately *N* = 278 (*n*_EG_ = 177; *n*_CG1_ = 49; *n*_CG2_ = 52) women remained in the final sample.

### Procedure

The present study – approved by an institutional ethics committee in Germany – was conducted from November 2020–March 2022. The recruiting process involved cooperation with gynaecologists, counsellors from special helpdesks, paediatrists, postnatal nurses, staff at daycare facilities for children, print media, social media and the homepage of the university where the study had been conducted. All interested women first took part in a telephone screening by the research team consisting of psychotherapists or clinically experienced psychologists at an advanced level. Women were informed that study participation was both anonymous and voluntary, the inclusion and exclusion criteria were checked and the women's current stress levels were established. Secondly, the online questionnaire was administered using Sosci survey (see below), finally the Diagnostic Interview for Mental Disorders (DIPS: Diagnostisches Interview bei psychischen Störungen; Margraf et al., 2021) was conducted online via the certified software Red Medical Systems™ (RedMedicalSystems, 2022) by a member of the research team or a student considering the level of distress in participants. All interviewers had completed DIPS training with certification and the results of all interviews (especially the diagnoses) were validated in intervision groups. The interviews differed in duration between 30 min and 6 h depending on the detail of the provided information. The participants received 30 euros for participating in the study.

### Instruments

In addition to socio‐demographic variables (e.g., age, level of education, presence of [living] children), data were collected for the examination group on the last pregnancy loss suffered as well as on the following potential predictors of depressive symptoms or depression: number of pregnancy losses, kind of pregnancy loss, coherent farewell from the deceased embryo/foetus (whether the woman was able to say goodbye in a manner that felt appropriate to her), medical explanation for the pregnancy loss, medical support, stress during pregnancy, occurrence of complications during pregnancy, stress immediately after the pregnancy loss, and history of psychotherapy/psychopharmacological treatments and other psychiatric therapies in the last 12 months. Additionally, changes in partnership and the mean score in the neuroticism scale of the short version of the Big Five Inventory (BFI‐K; Rammstedt & John, 2005) were established. Depressive symptoms were assessed using the State–Trait Anxiety‐Depression Inventory (STADI; Laux et al., 2013), the presence of a depressive disorder by the DIPS‐Interview (Margraf et al., 2021).

#### Short version of the big five inventory (BFI‐K; Rammstedt & John, 2005)

The BFI‐K enables the economic assessment of the personality dimensions extraversion, agreeableness, conscientiousness and neuroticism as well as openness. The psychometric properties of the BFI‐K (especially considering reliability and factorial validity) are acceptable (Kovaleva et al., 2013; Rammstedt & John, 2005). Below, only neuroticism will be regarded as it was strongly linked to depressive symptoms in women with early pregnancy loss (Farren et al., 2018). The internal consistency of the BFI‐K neuroticism subscale was acceptable (Cronbach's alpha = .74; Kovaleva et al., 2013).

#### State–trait anxiety‐depression inventory (STADI (Laux et al., 2013))

For assessing depressive symptoms, the State Depression Scale of the STADI was used in the present study. The self‐rating state questionnaire of this instrument consists of 20 items which can be answered on a 4‐point‐Likert scale, ranging between 1 = not at all (‘überhaupt nicht’), 2 = a little (‘ein wenig’), 3 = quite (‘ziemlich’) and 4 = very (‘sehr’). There is evidence of reliability (e.g., internal consistency for State Depression Scale (Cronbach's alpha) = .87) and satisfactory validity of the instrument, especially regarding factorial, convergent and discriminant validity (Laux et al., 2013).

#### Diagnostic interview for mental disorders (DIPS (Margraf et al., 2021))

The following sections of the semi‐structured diagnostic interview DIPS (fifth edition) were used to determine the current presence of various mental disorders (based on the DSM‐5; APA, 2013): Anxiety disorders, depressive disorders, obsessive‐compulsive disorder (OCD) and related mental disorders, trauma disorders and stress disorders, somatic symptom disorder and related mental disorders, sleep disorders, mental disorders associated with psychotropic drugs like substance use disorders (without pathological gambling). The frequency of sexual dysfunctions (which were only recorded as part of a screening) was also calculated. In order to record the point prevalence rates for adjustment disorders, a supplementary interview guide was created by the authors themselves, based on the sections of typical diagnostic interviews. The psychometric properties of the DIPS are good (Margraf et al., 2017).

### Statistical analysis

For the examination group, we performed a multiple binomial logistic regression analysis to predict the likelihood for the diagnosis of a current depressive episode based on the following predictors: age (in years; as metric variable), number of pregnancy losses (categories: one/>one; reference category: one), kind of pregnancy loss (categories: miscarriage, stillbirth; reference category: miscarriage), coherent farewell from the deceased embryo/foetus (categories: yes/no; reference category: yes), medical explanation for the pregnancy loss (categories: yes/no; reference category: yes), medical support (categories: yes/no; reference category: yes), stress during pregnancy (categories: yes/no; reference category: no), occurrence of complications during pregnancy (categories: yes/no; reference category: no), stress immediately after the pregnancy loss (metric variable; range of the corresponding rating scale: 0–100), history of psychotherapy/psychopharmacological treatments and other psychiatric therapies in the last 12 months (categories: yes/no; reference category: no), educational level (categories: university degree/general qualification for university entrance/no general qualification for university entrance; reference category: university degree), presence of (living) children (categories: yes/no; reference category: yes), changes of the own partnership (categories: yes/no; reference category: no) and the mean score in the neuroticism scale of the short version of the Big Five Inventory (BFI‐K; metric variable). According to preliminary analyses, the requirements for the multiple binomial logistic regression model were fulfilled: The dependent variable (current depressive episode: yes/no) was binary, the independent variables were either metric or categorical, the observations were independent, there were at least 10 cases per predictor as postulated by Peduzzi et al. (1996) and in only one case the studentized residual was above three suggesting an outlier; however, the corresponding leverage value was .04 indicating the absence of an outlier in the database according to a cut‐off score of .2 (see Huber, 1981). So, the corresponding data set had not been excluded from analyses. The linearity assumption was assessed by applying the Box‐Tidwell procedure (Box & Tidwell, 1962). In this context, the Bonferroni correction was chosen for all three terms in the statistical model (Tabachnick & Fidell, 2014). Linearity was given for all variables. Moreover, correlation between the independent predictor variables were lower than |*r*| = .70; thus, the results of the binomial logistic regression analysis were not confounded by multicollinearity. Regarding the multiple linear regression analysis, the requirements were fulfilled, too: Linearity was given because the predictor variables in the regression had a straight‐line association with the depression variable. The leverage values were ≤.20; thus, the presence of outliers in the database is unlikely according to a cut‐off score of .2 (see Huber, 1981). The regression model had no auto‐correlation as indicated by the value of the Durbin‐Watson statistic being near two (2.03). The correlation coefficients between the predictor variables were lower than |*r*| = .70; therefore, the results of the multiple linear regression analysis were not confounded by multicollinearity. The residuals were equally distributed as shown by scatterplots of the residuals against the predicted values of the State‐Depression sum score in the STADI. In addition, they were normally distributed as shown by the Shapiro–Wilk test (*p* = .543). For each predictor, the odds ratio, the corresponding 95% confidence interval (CI) and the corresponding (alpha‐adjusted) *p* value were presented. In addition, by using a multiple linear regression analysis it was examined whether the State‐Depression sum score in the STADI as measured in women with pregnancy loss within the previous 12 months could be predicted by the same independent variables as those chosen for the binomial logistic regression analysis (see above). Regression coefficients (*b*) with corresponding 95% CI and associated p values were calculated. In addition, standardized regression coefficients (*β*) were computed in order to demonstrate effects of variables which were independent of units. The SPSS version 29.0 for Windows (IBM Corp., Armonk, New York, USA) was selected for the statistical analyses and the significance level *α* = .05 was chosen. The Bonferroni correction was used in cases of multiple testing. All statistical tests were two‐tailed.

## RESULTS

### Characteristics of the sample

Table 1 shows the descriptive statistics of the sample.

**TABLE 1:** Descriptive statistics of the sample.

	EG (*n* = 177)
Age (Mean (SD))	34.30 (4.3)
Min; Max	24;44
Relationship status *n* (%)	
Single	5 (2.8)
In a relationship	49 (27.7)
Married	123 (69.5)
Separated	0 (.0)
Divorced	0 (.0)
Widowed	0 (.0)
Education level *n* (%)	
University degree	120 (67.8)
General qualification for university entrance	32 (18.1)
No general qualification for university entrance	24 (13.6)
n/a	1 (.6)
Employment *n* (%)	
Without current employment	16 (9.0)
Currently employed	161 (91.0)
Living children *n* (%)	
Yes	80 (45.2)
No	97 (54.8)
Treatment within the last 12 months yes/no; n_yes_ (%)	69/108 (39.0)
Psychotherapy	55/122 (31.1)
Psychiatric	14/163 (7.9)
Psychiatric drugs	22/155 (12.4)
Type of pregnancy loss^a^ *n* (%)	
Miscarriage	152 (85.9)
Stillbirth	25 (14.1)
Number of previous pregnancies (Mean (SD))	2.30 (1.7)
Min; Max	1;17
Number of previous losses (Mean (SD))	1.71 (1.5)
Min; Max	1;16
Ever fertility treatment *n* (%)	
No	133 (75.1)
Yes	44 (24.9)
Previous abortion *n* (%)	
No	175 (98.2)
Yes	2 (1.1)
Week of pregnancy at time of loss (Mean (SD))	13.63 (8.2)
Min; Max	3;39
Subjective psychological distress at time immediately after pregnancy loss (0‐100; Mean (SD))	79.72 (21.3)
Min; Max	10;100
Subjective psychological impairment immediately after pregnancy loss (0‐100; Mean (SD))	66.65 (27.3)
Min; Max	0;100
Time interval between loss and study participation in months (Mean (SD))	4.33 (2.9)
Min; Max	0;11

Abbreviations: %, percentages; EG, women with a miscarriage or stillbirth in the last 12 months; Max, maximum; Mean, mean value; Min, minimum; *n*, group size; n/a, not available; *n*_yes_, number of yes‐responses; SD, standard deviation.

^a^ Miscarriage: < 24th week of pregnancy and <500 g weight of the baby lost; stillbirth: ≥24th week of pregnancy or ≥500 g weight of the baby lost.

### Differences between women with versus without current major depressive episode after pregnancy loss

In a first step, differences between women with versus without a current major depressive episode after pregnancy loss regarding selected demographic variables and characteristics associated with pregnancy losses were analysed using univariate analyses (see Table 2). In addition, the table presents all comorbid diagnoses identified among participants with a current major depressive episode, where applicable.

**TABLE 2:** Differences between women with versus without a current major depressive episode after pregnancy loss regarding selected demographic variables and characteristics associated with the pregnancy loss.

Variables	Current major depressive episode (*N* = 172)		*p* value
	Yes (*n* = 17) (9.88%)	No (*n* = 155) (90.12%)	
Number of miscarriages or stillbirths *n* (%)			
1	9 (52.94%)	96 (61.94%)	.470^a^
>1	8 (47.06%)	59 (38.06%)	
Kind of pregnancy loss *n* (%)			
Miscarriage	13 (76.47%)	134 (86.45%)	.278^b^
Stillbirth	4 (23.53%)	21 (13.55%)	
Coherent farewell *n* (%)		(*n* = 153)	
No	7 (41.18%)	39 (25.49%)	.247^b^
Yes	10 (58.82%)	114 (74.51%)	
Medical explanation for the pregnancy loss *n* (%)			
No	12 (70.59%)	97 (62.58%)	.515^a^
Yes	5 (29.41%)	58 (37.42%)	
Medical support *n* (%)	(*n* = 16^f^)	(*n* = 152)	
No	11 (68.75%)	48 (31.58%)	.**003** ^**,^ ^a^
Yes	5 (31.25%)	104 (68.42%)	
Stress during pregnancy *n* (%)			
No	11 (64.71%)	96 (61.94%)	.823^a^
Yes	6 (35.29%)	59 (38.06%)	
Complications during pregnancy *n* (%)	(*n* = 16^f^)		
No	11 (68.75%)	115 (74.19%)	.766^b^
Yes	5 (31.25%)	40 (25.81%)	
Stress immediately after the pregnancy loss (range: 0–100) M (SD)	95.12 (7.29)	78.76 (20.90)	.**00012** ^***,^ ^c^
History of psychotherapy/psychiatry/psychotropic drugs in the last 12 months *n* (%)			
No	5 (29.41%)	100 (64.52%)	.**005** ^**,^ ^a^
Yes	12 (70.59%)	55 (35.48%)	
History of psychotherapy in the last 12 months *n* (%)			
No	7 (41.18%)	112 (72.26%)	.**008** ^**,^ ^a^
Yes	10 (58.82%)	43 (27.74%)	
History of psychiatry in the last 12 months *n* (%)			
No	13 (76.47%)	145 (93.55%)	.**036** ^*,^ ^b^
Yes	4 (23.53%)	10 (6.45%)	
History of psychotropic drugs in the last 12 months *n* (%)			
No	13 (76.47%)	137 (88.39%)	.**240** ^b^
Yes	4 (23.53%)	18 (11.61%)	
Age (in years) M (SD)	35.06 (5.06)	34.26 (4.24)	.502^c^
Educational level *n* (%)		(*n* = 154)	
University degree	10 (58.82%)	107 (69.48%)	.579^d^
General qualification for university entrance	4 (23.53%)	26 (16.88%)	
No general qualification for university entrance^e^	3 (17.65%)	21 (13.64%)	
Childlessness *n* (%)			
Children	7 (41.18%)	71 (45.81%)	.716^a^
No children	10 (58.82%)	84 (54.19%)	
Changes of partnership *n* (%)	(*n* = 15)	(*n* = 153)	
No	2 (13.33%)	39 (25.49%)	.365^b^
Yes	13 (86.67%)	114 (74.51%)	
BFI‐K scale means for neuroticism M (SD)	4.10 (.78)	3.33 (.86)	.**0005** ^***,^ ^c^
Psychiatric comorbidity in women with a current major depressive episode (*n* = 17)	–	–	**–**
Separation anxiety disorder	4 (23.53%)	–	**–**
Agoraphobia without history of panic disorder	1 (5.88%)	–	**–**
Social phobia	3 (17.65%)	–	**–**
Specific phobia	4 (23.53%)	–	**–**
Generalized anxiety disorder	2 (11.76%)	–	**–**
Persistent depressive disorder	2 (11.76%)	–	**–**
Posttraumatic stress disorder	9 (52.94%)	–	**–**
Somatic symptom disorder	1 (5.88%)	–	**–**
Insomnia	1 (5.88%)	–	**–**
Sexual dysfunction^f^	3 (18.75%)	–	**–**

*Note*: Significant findings are in bold. ^+^ *p* ≤ .10; *p ≤ .05; **p ≤ .01; ***p ≤ .001.

Abbreviations: BFI‐K, short version of the Big Five Inventory (Margraf et al., 2021); M, means; *N*, sample size; *n*, subgroup size; SD, standard deviation.

^a^ According to a chi‐square test for two‐by‐two cross tables.

^b^ According to Fisher's exact test for two‐by‐two cross tables.

^c^ According to a Mann–Whitney *U* test.

^d^ According to an exact Fisher Freeman Halton test for two‐by‐three cross tables.

^e^ General certificate of secondary education, certificate of secondary education, no graduation.

^f^ Data were available for only 16 cases.

Women after pregnancy loss and a current major depressive episode had received medical support significant less frequently (31.25%) than women with a miscarriage or stillbirth without a current major depressive episode (68.42%; χ^2^ = 8.778; df = 1; *p* = .003), had significantly higher stress scores immediately after the pregnancy loss (95.12 vs. 78.76; *Z* = −3.838; *p* = .00012), had significantly more often a history of psychotherapy or psychiatric treatment in the previous 12 months (70.59% vs. 35.48%; χ^2^ = 7.939; df = 1; *p* = .005) and had significantly higher BFI‐K scale means for neuroticism (4.10 vs. 3.33; *Z* = −3.461; *p* = .0005).

### Predictors for the presence of depressive disorders in women after pregnancy loss

Of the 14 independent variables in the logistic regression model, three (21.43%) were significant predictors of depressive disorders: low medical support (*p* = .041), stress immediately after the pregnancy loss (*p* = .027) and the BFI‐K scale means for neuroticism (*p* = .008), with none of them being statistically significant at the alpha‐adjusted significance level (.004). Low medical support was a risk factor, increasing the likelihood of a current major depressive episode (OR = 5.75; 95% CI: 1.07; 30.83); the same was true for stress immediately after the pregnancy loss (OR = 1.12; 95% CI: 1.01; 1.23) and the BFI‐K scale means for neuroticism (OR = 8.19; 95% CI: 1.74; 38.52). The corresponding results have been summarized in Table 3.

**TABLE 3:** Predictors for the presence of depressive disorders in women after pregnancy loss.

Variables	OR	95% CI for OR		*p* value
		Lower value	Upper value	
Number of miscarriages or stillbirths (categories: 1/>1; reference category: 1)	1.097	.213	5.645	.912
Kind of pregnancy loss (categories: miscarriage, stillbirth; reference category: miscarriage)	1.739	.108	28.059	.697
Coherent farewell (categories: yes/no; reference category: yes)	.850	.136	5.305	.862
Medical explanation for the pregnancy loss (categories: yes/no; reference category: yes)	1.211	.190	7.708	.839
Medical support (categories: yes/no; reference category: yes)	5.746	1.071	30.827	.**041** *
Stress during pregnancy (categories: yes/no; reference category: no)	.271	.036	2.043	.205
Complications during pregnancy (categories: yes/no; reference category: no)	.785	.086	7.150	.830
Stress immediately after the pregnancy loss (metric variable; range of the corresponding rating scale: 0–100)	1.117	1.013	1.232	.**027** *
History of psychotherapy/psychiatry in the last 12 months (categories: yes/no; reference category: no)	1.469	.249	8.668	.671
Age (in years; as metric variable)	1.060	.849	1.323	.606
Educational level (categories: university degree/general qualification for university entrance/no general qualification for university entrance; reference category: university degree)	–	–	–	–
General qualification for university entrance	.398	.032	4.999	.476
No general qualification for university entrance (general certificate of secondary education/certificate of secondary education/no graduation)	.147	.006	3.667	.243
Presence of living children (categories: yes/no; reference category: yes)	.802	.137	4.690	.807
Changes of partnership (categories: yes/no; reference category: no)	1.056	.113	9.886	.962
BFI‐K scale means for neuroticism (metric variable)	8.190	1.741	38.524	.**008** *

*Note*: Significant findings are in bold.

Abbreviations: BFI‐K, Short version of the Big Five Inventory (Margraf et al., 2021); CI, confidence interval; OR, odds ratio.

* *p* ≤ .05; none of them being statistically significant at the alpha‐adjusted significance level (.004).

### Predictors for the severity of depression in women after pregnancy loss

Of the 14 independent variables in the linear regression model, three (21.43%) significantly predicted the severity of depression: coherent farewell (*p* = .043), a history of psychotherapy or psychiatric treatment in the last year (*p* = .016) and the BFI‐K scale means for neuroticism (*p* < .001), with only the latter variable being significantly associated with the severity of depression at the alpha‐adjusted significance level (.004). A significantly positive association with the severity of depression was present for a history of psychotherapy or psychiatric treatment in the last year (*ß* = 2.67 (95% CI: .50; 4.84), standardized *β* = .19) and the BFI‐K scale means for neuroticism (*ß* = 2.75 (95% CI: 1.58; 3.93), standardized *β* = .35). In contrast, a significantly negative association with this variable was found for coherent farewell (*ß* = −2.38 (95% CI: −4.68; −.08), standardized *β* = −.15).

The corresponding results have been presented in Table 4.

**TABLE 4:** Predictors for the severity of depression in women after pregnancy loss.

Variables	Standardized *β*	*p* value	*ß*	95% CI for *ß*	
				Lower value	Upper value
Number of miscarriages or stillbirths (categories: 1/>1; reference category: 1)	−.013	.856	−.185	−2.198	1.828
Kind of pregnancy loss (categories: miscarriage, stillbirth; reference category: miscarriage)	.077	.332	1.560	−1.610	4.730
Coherent farewell (categories: yes/no; reference category: yes)	−.153	.**043** *	−2.378	−4.679	−.076
Medical explanation for the pregnancy loss (categories: yes/no; reference category: yes)	−.007	.925	−.102	−2.241	2.038
Medical support (categories: yes/no; reference category: yes)	−.138	.061	−1.975	−4.039	.089
Stress during pregnancy (categories: yes/no; reference category: no)	−.052	.488	−.736	−2.826	1.354
Complications during pregnancy (categories: yes/no; reference category: no)	−.038	.605	−.585	−2.816	1.645
Stress immediately after the pregnancy loss (metric variable; range of the corresponding rating scale: 0–100)	−.001	.987	.000	−.046	.046
History of psychotherapy/psychiatry in the last 12 months (categories: yes/no; reference category: no)	.189	.**016** *	2.671	.501	4.842
Age (in years; as metric variable)	−.006	.937	−.010	−.249	.230
Educational level (categories: university degree/general qualification for university entrance/no general qualification for university entrance (general certificate of secondary education/certificate of secondary education/no graduation); reference category: university degree)	−.080	.305	−.755	−2.204	.695
Presence of living children (categories: yes/no; reference category: yes)	−.048	.529	−.652	−2.693	1.388
Changes of partnership (categories: yes/no; reference category: no)	.003	.964	.052	−2.210	2.314
BFI‐K scale means for neuroticism (metric variable)	.352	**<.001** **	2.753	1.575	3.931

*Note*: Significant findings are in bold.

Abbreviations: BFI‐K, short version of the Big Five Inventory (Margraf et al., 2021); CI, confidence interval; *β*, regression coefficient Beta.

* *p* ≤ .05.

** Significant at the alpha‐adjusted significance level (.004).

## DISCUSSION

The main findings of the present study were as follows: Both univariate and multivariate analyses revealed that lower medical support, higher stress immediately after the pregnancy loss and higher neuroticism scores were significantly associated with an elevated risk for a current major depressive episode in women who had miscarriages or stillbirths within the last 12 months. In a multivariate linear regression analysis, a history of psychotherapy or psychiatric treatment within the past year and higher neuroticism scores were significantly associated with higher depressive symptom severity. In contrast, a coherent farewell process was significantly associated with lower depression scores. Moreover, there was a statistical tendency towards a negative association between medical support and severity of depressive symptoms. Beyond our primary findings, several candidate predictors showed no significant association with either a current major depressive episode or depression severity within the last year among women with pregnancy loss: number of miscarriages/stillbirths, type of pregnancy loss, medical explanation, stress during pregnancy, complications in the index pregnancy, age, educational level, childlessness and changes of partnership.

Our findings regarding the role of *medical support* as a predictor of depression in women with pregnancy losses align with two reviews suggesting professional support is a protective factor for depressive symptoms in patients with stillbirths (Burden et al., 2016; Westby et al., 2021). Additionally, they are consistent with a study demonstrating that low satisfaction with health care represents a risk factor for depressive symptoms in women with prior spontaneous abortions (deMontigny et al., 2020). Interestingly, we found a significant association between medical support and the occurrence of depressive disorders in our sample: Only 31.25% of women with a current major depressive episode received medical support after pregnancy loss, compared to 68.42% of women without a current major depressive episode. However, for depressive symptom severity, only a corresponding statistical trend was observed. Thus, medical support after a pregnancy loss seems to be more important as a protective factor regarding the occurrence of a major depressive episode than as a protective factor regarding depressive symptoms that do not necessarily indicate a depressive disorder. This finding is novel, because there is a lack of studies focused on medical support as a predictor of depressive disorders in women with pregnancy loss. One exception is a Chinese study by Sham et al. (2010); in this study, the authors examined the role of procedures following abortion as a risk factor for depressive disorders in women with miscarriages. However, the corresponding association failed to be statistically significant. Importantly, this predictor only partially captures the broader concept of medical support.

Regarding *stress immediately after the pregnancy loss*, this variable was assessed retrospectively in women with miscarriages or stillbirths within the last year and could be significantly associated with the occurrence of a current major depressive episode. The corresponding association with the intensity of depressive symptoms failed to be significant. The latter finding contrasts with one of the main results of the study by Kukulskiene and Zemaitiene (2022) who identified poorer physical and emotional well‐being immediately after miscarriage as risk factors for depressive symptoms in women who had at least one miscarriage in the past. In this context, it must be emphasized that we examined a mixed sample containing both women with miscarriages and women with stillbirths and that the pregnancy loss was within the last 12 months, whereas the years in which the last miscarriage in the Lithuanian sample of the study by Kukulskiene and Zemaitiene (2022) occurred ranged from 1993 to 2022. Thus, differences in the samples might have contributed to the afore‐mentioned discrepancies and a pronounced memory bias in the study by Kukulskiene and Zemaitiene due to a (partly) very long period between the last miscarriage and the investigations.

*Neuroticism* was both significantly associated with an increased risk for a current major depressive episode in women with pregnancy losses within the last 12 months and more severe depressive symptoms in our study. The former finding is new, whereas the latter result is in line with the narrative review by Farren et al. (2018) suggesting neuroticism to be a risk factor for depressive symptoms in women with early pregnancy loss. Our findings fit well to a twin study demonstrating that a considerable proportion of the genetic risk regarding major depressive disorder, manifesting through personality traits, is captured by neuroticism (Kendler & Myers, 2010). Additionally, a strong genetic link between neuroticism and depression was confirmed by a recent genome‐wide association study (Gupta et al., 2024). Thus, as a genetically influenced risk factor for depression, neuroticism may predispose vulnerable women to the development of a major depressive episode when confronted with a major life stressor, such as a pregnancy loss.

According to our study, an *incoherent farewell from the deceased embryo/foetus* was significantly associated with higher depression scores in women with miscarriages and stillbirths within the last 12 months. So far, this association has only been studied in women with stillbirths: Specially, not spending as much time with the baby as desired has been associated with more severe depressive symptoms in this group (for review, see Westby et al., 2021). Similarly, not seeing, holding or saying goodbye to the deceased baby was identified as a risk factor for depressive symptoms (Burden et al., 2016). Given that 85.9% of the women in our sample had miscarriages within the last year, our findings suggest that an incoherent farewell may also be associated with more severe depressive symptoms in women with miscarriages. However, the corresponding effect size was rather small and should therefore be interpreted with caution.

We also examined the role of a *history of psychotherapy/psychiatry in the last year* as a potential predictor for depression in women with pregnancy losses within the last 12 months. Among women with a current major depressive episode, 70.59% had such a history, compared to only 35.48% in women without a current major depressive episode following miscarriage or stillbirth within the last 12 months. Although this percentage difference was pronounced the corresponding effect size (*φ* = −.215) was small, and the corresponding result of a multivariate analysis failed to be significant. However, a history of psychotherapy or psychiatric treatment in the last year went along with significantly higher depression scores. This finding suggests that women with pregnancy loss within the last year who were also treated by a psychotherapist or a psychiatrist during this time period may represent a vulnerable subgroup with increased depressive symptoms, indicating a greater need for therapy.

### Methodological strengths and limitations

Methodological strengths of our study include the simultaneous examination of multiple potential predictors of both depressive symptoms and current major depressive disorder in women with miscarriages or stillbirths within the last 12 months. Additionally, we employed a well‐validated, semi‐structured clinical interview to assess depression. Moreover, we included the full spectrum of gestational ages at the time of pregnancy loss in our study and referred to the official definitions of miscarriages and stillbirths of the World Health Organization (WHO, 1975).

However, the major methodological limitation of our study is its cross‐sectional design; thus, prospective longitudinal studies are necessary to detect the direction of influence for the association between predictors and depression outcome in women with pregnancy loss. Additionally, the generalizability of our findings is limited by the overrepresentation of highly educated women in our sample. In addition, separate analyses for women with miscarriages and women with stillbirths could not be conducted because only 14.1% of women with pregnancy loss had stillbirths in our sample. It would also have been valuable to examine interactions between predictors of depression following pregnancy loss; however, the sample size was too small to allow for such analyses.

### Clinical implications

Our findings suggest that, in obstetric settings, early screening should include the assessment of satisfaction with medical support, marked stress immediately after the loss as well as neuroticism. Widely used instruments such as the EPDS (Edinburgh Postnatal Depression Scale; Bergant et al., 1998) or PHQ‐9 (Patient Health Questionnaire; Kroenke et al., 2001) can also be integrated into routine follow‐up and discharge calls. For prevention within obstetric care, services can prioritize timely, consistent and compassionate support (e.g., a designated contact person or a bereavement midwife), provide clear information and guidance on care options and facilitate a coherent farewell process aligned with women's preferences. Brief psychoeducation and crisis‐support offerings at the time of pregnancy loss may help reduce acute stress; where indicated, a stepped referral to specialist mental health care should follow. Tailored interventions may target specific risk profiles: for women with higher neuroticism, cognitive‐behavioural strategies that address rumination, emotion regulation and worry may be particularly relevant; for women already in psychotherapy or psychiatric care, close coordination with existing providers can ensure continuity; if the farewell process was incoherent, grief‐focused and meaning‐oriented interventions might be indicated. Embedding these components within a stepped‐care pathway with clearly defined referral thresholds for severe symptoms or major depressive episodes can support timely treatment.

## CONCLUSION

Our study demonstrated that the occurrence of a current major depressive disorder was significantly associated with lower medical support, higher stress immediately after the pregnancy loss and higher neuroticism scores in women with pregnancy loss within the last year. Additionally, depression severity was significantly linked to an incoherent farewell from the deceased embryo/foetus, a history of psychotherapy or psychiatric treatment within the last year and higher neuroticism scores. These predictors may be valuable for identifying high‐risk groups for depression among women with pregnancy losses. Recognizing these risk factors could facilitate the timely provision of specialized care interventions and mental health services, potentially preventing depressive symptoms from becoming chronic. Future prospective longitudinal studies should evaluate the effectiveness of such interventions and include larger numbers of stillbirth cases to improve representativeness. Furthermore, future research should investigate the extent to which a history of depressive disorders increases the risk of relapse in women after miscarriages or stillbirths. It will be also important to determine the specific time period during which this risk is heightened and to conduct similar analyses for anxiety symptoms. Additionally, future studies should distinguish between expected and unexpected pregnancy losses, as it can be hypothesized that unexpected miscarriages or stillbirths go along with increased depression risk. Finally, it would be also interesting to compare the risk of depression in women with missed abortion to that of women with spontaneous miscarriages.

## AUTHOR CONTRIBUTIONS

**Sarah M. Quaatz:** Conceptualization; data curation; investigation; methodology; project administration; supervision; validation; formal analysis; writing – original draft; writing – review and editing. **Roland Mergl:** Formal analysis; methodology; writing – original draft; writing – review and editing. **Franziska Bichlmayer:** Data curation; writing – original draft; writing – review and editing. **Helena Hoffmann:** Investigation; data curation; validation; writing – review and editing. **Kathryn Eichhorn:** Investigation; writing – review and editing. **Antje‐Kathrin Allgaier:** Conceptualization; data curation; investigation; methodology; project administration; supervision; validation; writing – review and editing. **Svenja Hoffmann:** Conceptualization; data curation; investigation; project administration; supervision; validation; writing – review and editing.

## CONFLICT OF INTEREST STATEMENT

The authors have no relevant financial or non‐financial interests to disclose.

## ETHICS STATEMENT

This study was approved by the Ethics Committee of the university where the study had been conducted and has been performed in accordance with the principles laid down in the current version of the Declaration of Helsinki. The privacy rights of human subjects have been observed.

## CONSENT TO PARTICIPATE

Informed and written consent to participate was obtained from all participants of this study.

## Supporting information


Table S1.

## Data Availability

The data sets generated and analysed during our study are not publicly available since the ethics approval does not include the publication of corresponding raw data. However, all data sets are available from the corresponding author of the manuscript upon reasonable request. The data sets will be de‐identified prior to the sharing with third parties.

## References

[bjc70032-bib-0001] APA . (2013). Diagnostic and statistical manual of mental disorders (5th ed.). American Psychiatric Association.

[bjc70032-bib-0002] Balle, S. R. , Nothelfer, C. , Mergl, R. , Quaatz, S. M. , Hoffmann, S. , Hoffmann, H. , Allgaier, A.‐K. , & Eichhorn, K. (2024). Depression after pregnancy loss: The role of the presence of living children, the type of loss, multiple losses, the relationship, and coping strategies. European Journal of Psychotraumatology, 15(1), 2386827. 10.1080/20008066.2024.2386827 39140607 PMC11328791

[bjc70032-bib-0003] Bergant, A. M. , Nguyen, T. , Heim, K. , Ulmer, H. , & Dapunt, O. (1998). German language version and validation of the Edinburgh postnatal depression scale. Deutsche Medizinische Wochenschrift (1946), 123(3), 35–40. 10.1055/s-2007-1023895 9472218

[bjc70032-bib-0004] Box, G. E. P. , & Tidwell, P. W. (1962). Transformation of the independent variables. Technometrics, 4(4), 531–550. 10.1080/00401706.1962.10490038

[bjc70032-bib-0005] Broen, A. N. , Moum, T. , Bödtker, A. S. , & Ekeberg, O. (2006). Predictors of anxiety and depression following pregnancy termination: A longitudinal five‐year follow‐up study. Acta Obstetricia et Gynecologica Scandinavica, 85(3), 317–323. 10.1080/00016340500438116 16553180

[bjc70032-bib-0006] Burden, C. , Bradley, S. , Storey, C. , Ellis, A. , Heazell, A. E. , Downe, S. , Cacciatore, J. , & Siassakos, D. (2016). From grief, guilt pain and stigma to hope and pride ‐ a systematic review and meta‐analysis of mixed‐method research of the psychosocial impact of stillbirth. BMC Pregnancy and Childbirth, 16, 9. 10.1186/s12884-016-0800-8 PMC4719709 26785915

[bjc70032-bib-0007] Campbell‐Jackson, L. , & Horsch, A. (2014). The psychological impact of stillbirth on women: A systematic review. Illness, Crisis and Loss, 22(3), 237–256. 10.2190/IL.22.3.d

[bjc70032-bib-0009] deMontigny, F. , Verdon, C. , Meunier, S. , & Dubeau, D. (2017). Women's persistent depressive and perinatal grief symptoms following a miscarriage: The role of childlessness and satisfaction with healthcare services. Archives of Women's Mental Health, 20(5), 655–662. 10.1007/s00737-017-0742-9 PMC5599434 28623418

[bjc70032-bib-0010] deMontigny, F. , Verdon, C. , Meunier, S. , Gervais, C. , & Coté, I. (2020). Protective and risk factors for women's mental health after a spontaneous abortion. Revista Latino‐Americana de Enfermagem, 28, e3350. 10.1590/1518-8345.3382.3350 32901768 PMC7478879

[bjc70032-bib-0011] Farren, J. , Jalmbrant, M. , Falconieri, N. , Mitchell‐Jones, N. , Bobdiwala, S. , Al‐Memar, M. , Parker, N. , Van Calster, B. , Timmerman, D. , & Bourne, T. (2022). Prognostic factors for post‐traumatic stress, anxiety and depression in women after early pregnancy loss: A multi‐centre prospective cohort study. BMJ Open, 12(3), e054490. 10.1136/bmjopen-2021-054490 PMC8889314 35232785

[bjc70032-bib-0012] Farren, J. , Mitchell‐Jones, N. , Verbakel, J. Y. , Timmerman, D. , Jalmbrant, M. , & Bourne, T. (2018). The psychological impact of early pregnancy loss. Human Reproduction Update, 24(6), 731–749. 10.1093/humupd/dmy025 30204882

[bjc70032-bib-0015] Gupta, P. , Galimberti, M. , Liu, Y. , Beck, S. , Wingo, A. , Wingo, T. , Adhikari, K. , Kranzler, H. R. , Program VMV , Stein, M. B. , Gelernter, J. , & Levey, D. F. (2024). A genome‐wide investigation into the underlying genetic architecture of personality traits and overlap with psychopathology. Nature Human Behaviour, 8(11), 2235–2249. 10.1038/s41562-024-01951-3 PMC11576509 39134740

[bjc70032-bib-0017] Hakulinen, C. , Elovainio, M. , Pulkki‐Råback, L. , Virtanen, M. , Kivimäki, M. , & Jokela, M. (2015). Personality and depressive symptoms: Individual participant meta‐analysis of 10 cohort studies. Depression and Anxiety, 32(7), 461–470. 10.1002/da.22376 PMC4605994 26014798

[bjc70032-bib-0018] He, L. , Wang, T. , Xu, H. , Chen, C. , Liu, Z. , Kang, X. , & Zhao, A. (2019). Prevalence of depression and anxiety in women with recurrent pregnancy loss and the associated risk factors. Archives of Gynecology and Obstetrics, 300(4), 1061–1066. 10.1007/s00404-019-05264-z 31485778

[bjc70032-bib-0019] Herbert, D. , Young, K. , Pietrusińska, M. , & MacBeth, A. (2022). The mental health impact of perinatal loss: A systematic review and meta‐analysis. Journal of Affective Disorders, 297, 118–129. 10.1016/j.jad.2021.10.026 34678403

[bjc70032-bib-0020] Hu, Y. , Tang, R. , Li, X. , Wang, X. , Ma, H. , Heianza, Y. , Qi, L. , & Liang, Z. (2024). Spontaneous miscarriage and social support in predicting risks of depression and anxiety: A cohort study in UK biobank. American Journal of Obstetrics and Gynecology, 231(6), 655.e1–655.e9. 10.1016/j.ajog.2024.03.045 38588963

[bjc70032-bib-0021] Huber, P. J. (1981). Robust statistics. John Wiley.

[bjc70032-bib-0022] Kendler, K. S. , & Myers, J. (2010). The genetic and environmental relationship between major depression and the five‐factor model of personality. Psychological Medicine, 40(5), 801–806. 10.1017/s0033291709991140 19732485

[bjc70032-bib-0023] Kovaleva, A. , Beierlein, C. , Kemper, C. J. , & Rammstedt, B. (2013). Psychometric properties of the BFI‐K: A cross‐validation study. International Journal of Educational and Psychological Assessment, 13(1), 34–50.

[bjc70032-bib-0024] Kroenke, K. , Spitzer, R. L. , & Williams, J. B. (2001). The PHQ‐9: Validity of a brief depression severity measure. Journal of General Internal Medicine, 16(9), 606–613. 10.1046/j.1525-1497.2001.016009606.x PMC1495268 11556941

[bjc70032-bib-0025] Kukulskienė, M. , & Žemaitienė, N. (2022). Postnatal depression and post‐traumatic stress risk following miscarriage. International Journal of Environmental Research and Public Health, 19(11), 6515. 10.3390/ijerph19116515 PMC9180236 35682100

[bjc70032-bib-0026] Laux, L. , Hock, M. , Bergner‐Köther, R. , Hodapp, V. , & Renner, K.‐H. (2013). Das state‐trait‐angst‐depressions‐Inventar: STADI. Manual. Hogrefe.

[bjc70032-bib-0028] Margraf, J. , Cwik, J. C. , Pflug, V. , & Schneider, S. (2017). Strukturierte klinische Interviews zur Erfassung psychischer Störungen über die Lebensspanne ‐ Gütekriterien und Weiterentwicklungen der DIPS‐Verfahren. Zeitschrift Fur Klinische Psychologie Und Psychotherapie, 46(3), 176–186.

[bjc70032-bib-0029] Margraf, J. , Cwik, J. C. , von Brachel, R. , Suppiger, A. , & Schneider, S. (2021). DIPS Open Access 1.2: Diagnostic Interview for Mental Disorders. [DIPS Open Access 1.2: Diagnostisches Interview bei psychischen Störungen]. Mental Health Research and Treament Center, Ruhr‐Universität Bochum.

[bjc70032-bib-0030] Mergl, R. , Quaatz, S. M. , Lemke, V. , & Allgaier, A.‐K. (2024). Prevalence of depression and depressive symptoms in women with previous miscarriages or stillbirths – A systematic review. Journal of Psychiatric Research, 169, 84–96. 10.1016/j.jpsychires.2023.11.021 38006823

[bjc70032-bib-0031] Mutiso, S. K. , Murage, A. , & Mukaindo, A. M. (2018). Prevalence of positive depression screen among post miscarriage women‐ a cross sectional study. BMC Psychiatry, 18(1), 32. 10.1186/s12888-018-1619-9 PMC5799918 29402255

[bjc70032-bib-0032] Mutiso, S. K. , Murage, A. , & Mwaniki, A. M. (2019). Factors associated with a positive depression screen after a miscarriage. BMC Psychiatry, 19(1), 8. 10.1186/s12888-018-1991-5 PMC6323848 30616554

[bjc70032-bib-0033] Obi, S. N. , Onah, H. E. , & Okafor, I. I. (2009). Depression among Nigerian women following pregnancy loss. International Journal of Gynaecology and Obstetrics, 105(1), 60–62. 10.1016/j.ijgo.2008.11.036 19111302

[bjc70032-bib-0034] Peduzzi, P. , Concato, J. , Kemper, E. , Holford, T. R. , & Feinstein, A. R. (1996). A simulation study of the number of events per variable in logistic regression analysis. Journal of Clinical Epidemiology, 49(12), 1373–1379. 10.1016/s0895-4356(96)00236-3 8970487

[bjc70032-bib-0035] Quenby, S. , Gallos, I. D. , Dhillon‐Smith, R. K. , Podesek, M. , Stephenson, M. D. , Fisher, J. , Brosens, J. J. , Brewin, J. , Ramhorst, R. , Lucas, E. S. , McCoy, R. C. , Anderson, R. , Daher, S. , Regan, L. , Al‐Memar, M. , Bourne, T. , MacIntyre, D. A. , Rai, R. , Christiansen, O. B. , … Coomarasamy, A. (2021). Miscarriage matters: The epidemiological, physical, psychological, and economic costs of early pregnancy loss. Lancet, 397(10285), 1658–1667. 10.1016/s0140-6736(21)00682-6 33915094

[bjc70032-bib-0036] Rammstedt, B. , & John, O. P. (2005). Kurzversion des big five inventory (BFI‐K): Entwicklung und Validierung eines ökonomischen Inventars zur Erfassung der fünf Faktoren der Persönlichkeit [short version of the big five inventory (BFI‐K): Development and validation of an economic inventory for assessment of the five factors of personality]. Diagnostica, 51(4), 195–206. 10.1026/0012-1924.51.4.195

[bjc70032-bib-0037] RedMedicalSystems . (2022). Red Medical Systems. Red Medical Systems GmbH.

[bjc70032-bib-0038] Sham, A. , Yiu, M. , & Ho, W. (2010). Psychiatric morbidity following miscarriage in Hong Kong. General Hospital Psychiatry, 32(3), 284–293. 10.1016/j.genhosppsych.2009.12.002 20430232

[bjc70032-bib-0041] Tabachnick, B. G. , & Fidell, L. S. (2014). Using multivariate statistics. Pearson. https://elibrary.pearson.de/book/99.150005/9781292034546

[bjc70032-bib-0042] UNICEF, WHO, WBG, UN . (2020). A neglected tragedy: The global burden of stillbirths 2020 ‐ Estimates developed by the UN Inter‐agency group for child mortality estimation. https://www.unicef.org/reports/neglected‐tragedy‐global‐burden‐of‐stillbirths‐2020

[bjc70032-bib-0043] Wang, T. T. , Liu, Y. L. , Hou, Y. , Li, J. P. , & Qiao, C. (2023). The risk factors of progestational anxiety, depression, and sleep disturbance in women with recurrent pregnancy loss: A cross‐sectional study in China. Frontiers in Psychology, 14, 1116331. 10.3389/fpsyg.2023.1116331 PMC10102375 37063554

[bjc70032-bib-0044] Westby, C. L. , Erlandsen, A. R. , Nilsen, S. A. , Visted, E. , & Thimm, J. C. (2021). Depression, anxiety, PTSD, and OCD after stillbirth: A systematic review. BMC Pregnancy and Childbirth, 21(1), 782. 10.1186/s12884-021-04254-x PMC8600867 34794395

[bjc70032-bib-0045] WHO . (1992). The ICD‐10 classification of mental and behavioural disorders: Clinical descriptions and diagnostic guidelines. World Health Organization.

[bjc70032-bib-0046] WHO . (1975). Manual of the international statistical classification of diseases, injuries, and causes of death: Based on the recommendations of the ninth revision conference, 1975, and adopted by the Twenty‐ninth World Health Assembly 1975. https://apps.who.int/iris/handle/10665/40492

[bjc70032-bib-0047] Zhang, Y. , Feng, M. , Gao, Y. , Zhang, M. , & Zhang, Z. (2024). Depression outcome in women with recurrent spontaneous abortion: A systematic review and meta‐analysis. European Journal of Obstetrics, Gynecology, and Reproductive Biology, 300, 54–62. 10.1016/j.ejogrb.2024.06.044 38986273

