# Supplementary material for: Predictors of depressive symptoms and depression in women with previous pregnancy loss
Source: Br J Clin Psychol. 2025 Dec 28;65(2):420–38. doi: 10.1111/bjc.70032 (PMC13159768; doi:10.1111/bjc.70032)
Supplement: Supplementary file 1 — Table S1. [file BJC-65-420-s001.docx]

Research article

**Predictors of depressive symptoms and depression in women with previous pregnancy loss**

**Online Resource**

**Table 1: Overview of previous studies investigating predictors of depressive disorders or symptoms after pregnancy loss**

| **Author and**  **year of publication** | **Title** | **Country** | **Sample characteristics** | | | **Method** | | | **Predictors** | **Additional information** |
| --- | --- | --- | --- | --- | --- | --- | --- | --- | --- | --- |
|  |  |  | ***Sample size*** | ***Mean age (years)*** | ***Time of loss / duration of the pregnancy*** | ***Kind of pregnancy*** | ***Instrument for the assessment of depressive symptoms or disorder***  (longitudinal studies with measurement time of depression / predictors) | ***Control group*** |  |  |
| [15]^2^ | Spontaneous miscarriage and social support in predicting risks of depression and anxiety: a cohort study in UK Biobank | United Kingdom | *N* = 179,900  Number of spontaneous miscarriages (NSM):  0: *n* = 136,645 (76.3%)  1: *n* = 30,670 (17.1%)  2: *n* = 7,584 (4.2%)  ≥3: *n* = 3,968 (2.2%) | NSM = 0:  *M*_age_ = 57.2  *SD* = 7.8  NSM = 1:  *M*_age_ = 56.5  *SD* = 8.0  NSM = 2:  *M*_age_ = 56.2  *SD* = 8.1  NSM ≥ 3:  *M*_age_ = 55.8  *SD* = 8.2 | n.s. | Spontaneous miscarriage | Diagnoses obtained from hospital inpatient records: ICD-10 codes F32 (depressive episode), F33 (recurrent depressive disorder), F34 (persistent mood disorders), F38 (other mood disorders), and F39 (unspecified mood disorder) for depression  Median follow-up of 12.3 years | yes | **History of spontaneous miscarriage***  **Per addition decrease of social support***  **Joint effect***  (according to multivariable adjusted relative risks)  Only NSM = 1 (reference = 0) (according to adjusted hazard ratios):  **Age <57 years***  **White ethnicity***  **Townsend deprivation index ≥ median***  **No hypertension at baseline***  **No diabetes at baseline***  **No cardiovascular disease at baseline***  **No cancer at baseline***  **No live births***  **No history of gestational diabetes mellitus***  **No history of hypertensive disorders of pregnancy***  **BMI ≥ 25***  **Diet score <2***  **Regular physical activity***  **Never smoking***  Alcohol consumption  Age ≥57 years  Non-white ethnicity  Townsend deprivation index < median  hypertension at baseline  diabetes at baseline  cardiovascular disease at baseline  cancer at baseline  live births  history of gestational diabetes mellitus  history of hypertensive disorders of pregnancy  BMI < 25  Diet score ≥2  Irregular physical activity  Previous smoking  Current smoking | 4,939 incident cases of depression (2.75%) during a median follow-up of 12.3 years |
| [23]^1^ | The risk factors of progestational anxiety, depression, and sleep disturbance in women with recurrent pregnancy loss: A cross-sectional study in China | China | *N =* 951  Women with recurrent PL: *n* = 663  Women with one history of PL: *n* = 124  Women without history of previous PL: *n* = 74 | Women with recurrent PL:  *M*_age_ = 32.83 years  *SD* = 4.05  Women with one history of PL:  *M*_age_ = 32.97 years  *SD* = 4.09  Women without history of previous PL:  *M*_age_ = 33.07 years  *SD* = 4.51 | n.s. | Spontaneous miscarriage (group 1: ≥2; group 2: 1 in the past; group 3: 0) | Occurrence of depressive symptoms measured by using the sum scores of the CESD (Chinese version) (cut-off score: ≥ 16) | yes | **Women with recurrent PL (*n* = 663):**  **number of miscarriages ≥4*** (also after adjustment for covariables)  **Low monthly household income*** (also after adjustment for covariables)  **interval since the last miscarriage**  **<six months*** (also after adjustment for covariables)  **sleep disturbance*** (also after adjustment for covariables)  **state anxiety*** (also after adjustment for covariables)  **trait anxiety*** (also after adjustment for covariables)  age  BMI score  educational level  length of marriage  PL after 14 weeks (yes/no)  live birth (yes/no)  active smoking (yes/no)  passive smoking (yes/no)  alcohol drinking (yes/no) | Women with recurrent PL had significantly higher CESD sum scores than the control groups:  Women with recurrent PL:  *M* = 12.76  *SD* = 8.63  Women with one history of PL:  *M* = 11.69  *SD* = 8.92  Women without history of previous PL:  *M* = 10.22  *SD* = 6.80 |
| [16]^1^ | Depression after pregnancy loss: the role of the presence of living children, the type of loss, multiple losses, the relationship quality, and coping strategies | Germany | *N* = 172  *n*_miscarriage_ = 137  (79.7%)  *n*_stillbirth_ = 35  (20.3%) | *M*_age_ = 34.45  *SD* = 4.39 | week of pregnancy at time of loss:  *M* = 14.71  *SD* = 8.87 | miscarriage or stillbirth within the last  12 months | depressive scores via PHQ-D | no | **stillbirth*** (type of PL)  **childlessness***  **self-blame/ emotional avoidance***  **short time interval between last PL and date of interview***  multiple PL  PFB tenderness  PFB dispute behavior  PFB commonality  social support  acceptance and positive reframing  active problem solving  substance consumption  humor  distraction  religion  negative emotionality  age  (results according to multiple linear hierarchical regression analyses adjusted for age and the time interval between the last PL and the date of the interview) |  |
| [9]^1^ | Postnatal Depression and Post-Traumatic Stress Risk  Following Miscarriage | Lithuania | *N* = 839 | *M*_age_ = 33.34  *SD* = 5.46 | n.s. | miscarriage (at least one) in the past | postnatal depressive Risk via EPDS cut-off scores | no | **younger age (<35 years)***  **no higher education***  **worse emotional wellbeing before pregnancy***  **worse physical well-being immediately after miscarriage***  **worse emotional well-being immediately after miscarriage***  **impaired relationship with one´s body***  **less support from family/close friends***  type of miscarriage (early vs. late)  marital status (unmarried/married)  number of miscarriages  number of children born in the family (0/≥1)  (results according to an unadjusted multivariate binary logistic regression analysis) | Increased risk of postnatal depression (EPDS overall score ≥ 10):  59.1%  High risk of postnatal depression (EPDS overall score ≥ 12): 48.9%  self-reported severity of depression |
| [7]^2^ | Prognostic factors for post-traumatic stress, anxiety and depression in women after early pregnancy loss: a multi-centre prospective cohort study | United Kingdom | *N* = 737  *n*_Depression_ = 492 (66.76%) | *M*_age_ = 34  *SD* = 5 | PL before 20 weeks | PL   - miscarriage (including molar pregnancy) - ectopic pregnancy - resolved pregnancy of unknown location | depressive symptoms via HADS one month after PL | no | **psychiatric history***  **previous losses***  **ethnicity (other than Asian, black or white)***  **previous termination of pregnancy***  time to conceive (>1 year)  final diagnosis (miscarriage vs. ectopic vs. other)  final management (surgical, medical, conservative)  fetal heart on previous ultrasound imaging (miscarriage only)  age  highest level of education  overnight admission  presence of previous children  number of days to diagnosis  IVF pregnancy  Gestational age  (results according to unadjusted univariate binary logistic regression analyses) |  |
| [6]^1^ | Protective and risk factors  for women’s mental health after a spontaneous abortion | Canada | *N* = 231 | *M*_age_ = 30.74  *SD* = 4.60 | 3-20 weeks | spontaneous abortions in the past four years | depressive symptoms via EPDS (French version) | no | **less time since miscarriage (<six months)* (**also after adjustment for covariables)  **lower family income (<50,000 CAD)* (**also after adjustment for covariables)  **being an immigrant*** (only according to an ANOVA)  **childlessness*** (only according to an ANOVA)  **low quality of the conjugal relationship* (**also after adjustment for covariables)  **low satisfaction with health care* (**also after adjustment for covariables)  age at the time of the spontaneous abortion  schooling  gestational age of the foetus  number of spontaneous abortions | prevalence of possible depression:  55% (EPDS overall score ≥ 10) |
| [17]^1^ | Factors associated with a positive depression screen after a miscarriage | Kenia | *N* = 182 | *M*_age_ = n.s.  *SD* = n.s. | n.s. | miscarriage | depressive symptoms via EPDS | no | **increasing age* low educational level***  **increasing gestational age at miscarriage***  **being single***  **assisted mode of conception* prior miscarriage***  planning of the pregnancy  social support  others aware of pregnancy  mode of treatment of the miscarriage  prior pregnancy outcome  (results according to an unadjusted multivariate binary logistic regression analysis) | positive depression screen: 34.1% |
| [8]^1^ | Prevalence of depression and anxiety in women with recurrent  pregnancy loss and the associated risk factors | China | *N* = 1138  *n*_RecurrentPL_ = 782  *n*_1PL_ = 218  *n*_CG_ = 138 (no history of pregnancy loss) | Recurrent PL:  *M*_age_ = 31.42  *SD* = 4.63  1 PL:  *M*_age_ = 31.48  *SD* = 4.82  CG:  *M*_age_ = 31.91  *SD* = 5.51 | PL before 24^th^ weeks´gestation | recurrent PL with two or more miscarriages  including biochemical pregnancy loss and confirmed intrauterine pregnancy loss | depressive symptoms via SDS | yes | **Lower education level*** (also after adjustment for covariables)  **multiple pregnancy losses (≥3 versus 2)*** (also after adjustment for covariables)  **lower household income (<10,000 yuan)* (according to univariate unadjusted analysis)**  **history of induced abortion* (according to univariate unadjusted analysis)**  **no previous live birth* (according to univariate unadjusted analysis)**  Age  Age of the spouse  BMI  Education of the spouse  Duration of marriage (years) (<3 versus ≥3)  Pregnancy loss > 12 weeks  (the latter six variables were only independent variables in univariate unadjusted statistical tests) | prevalence of mild/moderate  depression:  Recurrent PL = 7.2/2.7%  1 PL = 8.7/1.8%  CG = 3.6/2.2% |
| [22]^1^ | Women’s persistent depressive and perinatal grief symptoms following a miscarriage: The role of childlessness and satisfaction with healthcare services | Canada | *N* = 245 | *M*_age_ = 31  (18–24 years: 21 (8.64%); 25–34 years: 167 (68.72%); ≥35 years: 55 (22.63%) (n=243) | PL during the first 27 weeks of pregnancy | at least  one miscarriage in the past six years | depressive symptoms via EPDS (French version) | no | **childlessness*** (also for longer duration of depressive symptoms)  **time since miscarriage*** (0-6 months > 7-12 months; 0-6 months > more than two years)  dissatisfaction with healthcare services  (according to unadjusted analyses) |  |
| [20]^2^ | Psychiatric morbidity following miscarriage in Hong Kong | China | T1 (immediately after miscarriage):  *N* = 181  T2 (3 months later):  *N* = 161 | *M*_age_ = 33.7  *SD* = 5.9 | PL before the 23rd completed week of gestation | miscarriage | depressive disorder via SCID,  GHQ-12  (immediately after loss and after three months), if the GHQ result was above the cutoff (4),  depressive disorder via SCID (after three months) was measured | no | **younger age*** (also after adjustment for covariables)  **history of infertility*** (also after adjustment for covariables)  **past history of depression*** (also after adjustment for covariables)  **The following predictors according to unadjusted statistical tests:**  **past psychiatric history***  **past suicidal history***  **work-related problems***  marital status  educational level  vocational activity  total monthly family income  number of household members  number of living children  number of previous pregnancy losses  number of previous induced abortion  past history of domestic violence  family history of mental illness  smoking  alcohol drinking  substance use  gestational age at time of miscarriage  attitude towards pregnancy (planned/unplanned)  procedure following abortion  recent health/death related issue  recent marital/partner relationship problem  recent financial problem  recent housing issue  recent legal/crime  other recent life events (including reproductive events among friends/relatives)  relationship with spouse,  relationship with maiden relatives | prevalence of depressive disorder:  9.94% |
| [18]^1^ | Depression among Nigerian women following pregnancy loss | Nigeria | *N* = 202 | *M*_age_ = 26.8  *SD* = 5.37 | 65.84% <20 weeks gestation | PL over one-year period   - involuntary PL - PL included spontaneous abortions, ectopic pregnancies, fetal deaths, and stillbirths | depressive symptoms  via SDS | no | **previous PL***  **childlessness* (also significant according to multiple binary logistic regression analysis)**  **gestational age at PL ≥ 20 weeks (also significant according to multiple binary logistic regression analysis)**  **loss of male foetus***  **being married***  age  education status  All statistical tests were unadjusted. |  |
| [19]^2^ | Predictors of anxiety and depression following pregnancy termination: A longitudinal five-year follow-up study | Norway | *N* = 120 | miscarriage  *M*_age_ = 30.1  *95% KI* = 28.2-31.9 (*n* = 40) (T1)  induced abortion  *M*_age_ = 27.7  *95% KI* = 26.2-29.3 (*n* = 80) (T1) | miscarriage:  M_length_ = 10.5 weeks  *95% KI* = 9.4-11.5  induced abortion: M_length_ = 9.6 weeks  *95% KI* = 9.3-9.9 | miscarriage and induced abortion | Depressive scores and depressive disorder (ICD-10 evaluation) via HADS and Life Events Scale ten days (T1), six months (T2), two years (T3), and five years (T4) after the event, ICD-10 interviews | yes | **For depression six months after a miscarriage (according to unadjusted multivariate linear regression analysis):**  **recent life events (3 or more) the previous half year***  **poorer former psychiatric health***  age  length of pregnancy  number of previous induced abortions  number of previous miscarriages  number of children  marital status  education  vocational activity  former psychiatric health  information regarding current pregnancy |  |

*Notes:*

^1^ cross-sectional study; ^2^ longitudinal study; *significant predictor, significant findings are in bold, too;

*CG* = control group; *CESD* = Center for Epidemiological Survey, Depression Scale [41]; *EPDS* = Edinburgh Postnatal Depression Scale [42]; *GHQ-12* = 12-Item General Health Questionnaire [43]; *HADS* = Hospital Anxiety Depression Scale [44]; *ICD-10* = International Statistical Classification of Diseases and Related Health Problems [14]; *IVF* = in vitro fertilization; *M* = Mean; *M*_age_ = mean age; *N/n* = sample size; n.s. = not specified; *PFB* = Partnerschaftsfragebogen (original title; in English: Relationship questionnaire) [45]; *PHQ-D* = Patient Health Questionnaire, German version [46]; *PL* = pregnancy loss; *SCID* = Structured Clinical Interview for DSM-IV [47]; *SD* = standard deviation; *SDS* = Zung Self-Rating Depression Scale [48].
